# Pseudogenes of annexin A2, novel prognosis biomarkers for diffuse gliomas

**DOI:** 10.18632/oncotarget.22197

**Published:** 2017-10-31

**Authors:** Shuang Li, Hecun Zou, Ying-Ying Shao, Ying Mei, Yu Cheng, Dong-Li Hu, Zhi-Rong Tan, Hong-Hao Zhou

**Affiliations:** ^1^ Department of Clinical Pharmacology, Xiangya Hospital, Central South University, Changsha 410008, China; ^2^ Institute of Clinical Pharmacology, Central South University, Hunan Key Laboratory of Pharmacogenetics, Changsha 410078, China; ^3^ Institute of Life Sciences, Chongqing Medical University, Chongqing 400016, China

**Keywords:** pseudogene, diffuse gliomas, biomarkers, ANXA2, ANXA2P1 ANXA2P2 ANXA2P3

## Abstract

Diffuse gliomas is a kind of common malignant primary brain tumor. Pseudogenes have multilayered biological function in the progression of human cancers. In this study, Differentially Expressed Pseudogenes (DEPs) between glioblastomas and non-tumor controls were found by bioinformatics analysis, of which the annexin A2 pseudogenes (ANXA2P1, ANXA2P2 and ANXA2P3) were significantly up-regulated, along with the parent gene annexin A2 (ANXA2*)*. Among four glioblastoma subtypes, ANXA2P1 and ANXA2P2 were preferentially expressed in mesenchymal subtype and less expressed in proneural subtype. Meanwhile, Pearson’s correlation analysis revealed that the expression level of ANXA2 was positively correlated with ANXA2 pseudogenes expression. Then, the expression patterns of ANXA2 and its pseudogenes were validated in diffuse glioma specimens (n=99) and non-tumor tissues (n=12) by quantitative real-time PCR (qRT-PCR). Additionally, Kaplan–Meier analysis revealed that highly expressed ANXA2 and annexin A2 pseudogenes were associated with the poor survival outcome of glioma patients. Cox regression analyses suggested that ANXA2, ANXA2P1 and ANXA2P2 were the independent prognosis factors for gliomas. Furthermore, down-regulation of ANXA2 and ANXA2 pseudogenes might contribute to the improvement of patients’ survival who received chemotherapy and radiotherapy. These results demonstrated that ANXA2 pseudogenes and ANXA2 could be used as the novel biomarkers for diagnosis, prognosis and target therapy of gliomas.

## INTRODUCTION

Based on a combination of histologic diagnostics with molecular features, the newest World Health Organization (WHO) classification of Central Nervous System (CNS) tumors presents a restructuring of the gliomas. Astrocytic tumors and oligodendrogliomas with WHO grade II and grade III, glioblastomas (GBM) with grade IV, are diffuse gliomas [1]. Diffuse gliomas is the common malignant and aggressive human brain tumor that has poor survival, especially for GBM. The median survival duration of GBM is less than 15 months, despite the comprehensive treatments including surgical resection and adjuvant chemoradiotherapy [2]. Moreover, due to the lack of precise early diagnosis and meaningful treatment, a great part of patients with gliomas are suffering from a poor quality of life, such as a variety of cognitive deficits [3] and psychosocial issues [4, 5]. Thus, the identification of novel candidate biomarkers for diagnosis, prognosis and treatment is urgently needed.

The prevalence of pseudogenes in human genome has been highlighted following the completion of Human Genome Project ENCODE (Encyclopedia of DNA Elements) and GENCODE [6, 7]. Although the pseudogenes had been regarded as “genomic fossil” or “junk genes” early [8, 9], recent studies have demonstrated that pseudogenes have multilayered biological function in processes of pathology and physiology [10], especially in human cancers progression [11]. For instance, the expressions of numerous pseudogenes were tissue- and cancer-specific [12]. Particularly, pseudogenes may play regulatory roles in both transcription and post-transcription of tumor cells. One of the pseudogenes named PTENP1, down-expressed in Hepatocellular carcinoma (HCC) cells and showed the anti-carcinoma properties due to its ability to regulate cellular levels of its parent gene (PTEN) [13]. Meanwhile, the expression of PTENP1 is correlated with PTEN in clear-cell renal cell carcinoma (ccRCC) and prostate cancer, and PTENP1 exert a growth-suppressive role by functions as a competing endogenous RNA (ceRNA) [14–16]. In addition, PTENP1 had two encoded antisense RNA (asRNA) isoforms, alpha and beta, which is biologically active by regulating PTEN transcription and PTEN mRNA stability [17]. However, the expression pattern and potential function of pseudogenes in diffuse glioma are still unclear.

In the present study, we aim to find certain pseudogenes as suitable biomarkers for diagnostic or therapeutic strategies in diffuse gliomas patients. Firstly, we identified differentially expressed pseudogenes based on gene expression profiling, and selected certain pseudogenes with significantly differential expression in diffuse gliomas. Additionally, to reveal the underlying clinical significance, we studied the correlations of these pseudogenes’ expression with glioma patients’ Overall Survival (OS) and chemoradiotherapy outcome.

## RESULTS

### The characters of pseudogenes expression in malignant glioma and non-tumor controls

**Figure 1:**
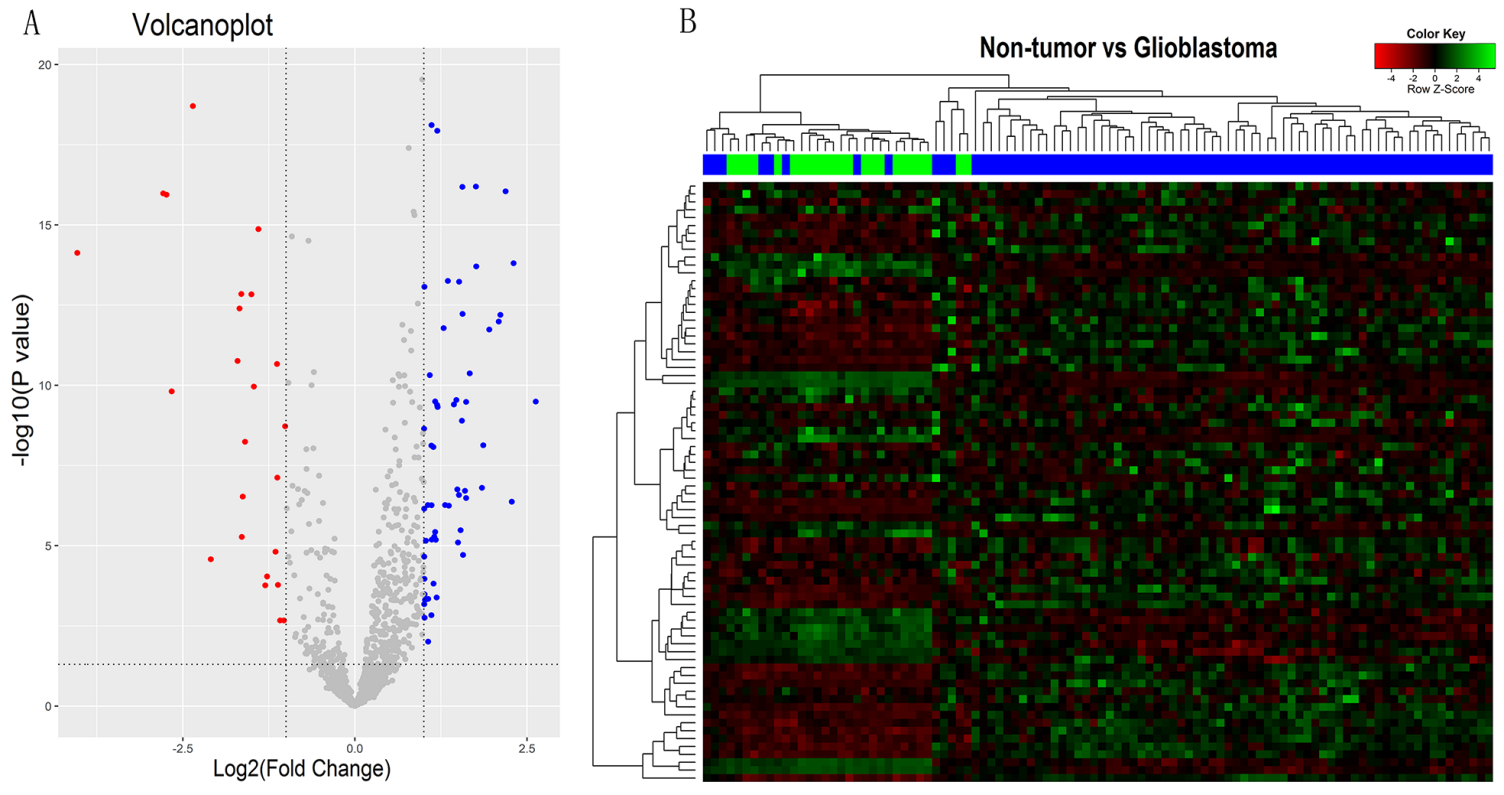
The pseudogenes expression characters in malignant gliomas and non-tumors controls **(A)** Volcano plots showing the difference of pseudogenes expression between malignant glioma and non-tumor controls in GSE4290. The blue and the red points represent the upregulated pseudogenes and the downregulated pseudogenes in malignant glioma, respectively. |logFC|>1 and P<0.05 set as the criteria outlined. **(B)** Heat-map for the values of the different expression pseudogenes (DEPs). The green-shaded areas clustered as the high-expressed pseudogenes, and the magenta-shaded areas indicate the lower expression pseudogenes. Sample dendrogram shows the relatedness among samples. The blue and green bars represent the glioblastoma samples and non-tumor controls, respectively.

Based on the Affymetrix annotation of the probe sets and the Refseq annotations of pseudogenes, we sought out 1018 probe sets (corresponding to 768 pseudogenes) that were represented on the Affymetrix HG-U133 Plus 2.0 arrays (Supplementary Table 1). Using bioinformatics analysis, we identify differentially expressed pseudogenes (DEPs) between 77 glioblastoma samples and 23 normal controls within GSE4290 dataset. As shown in the volcano plots (Figure 1A), a total of 73 DEPs were identified according to the criteria outlined (|logFC|>1; P<0.05), including 51 upregulated pseudogenes and 22 downregulated pseudogenes. Cluster analysis was performed with gene expression values for DEPs, and the heat-map was shown in Figure 1B. Interestingly, among the DEPs, we found that annexin A2 pseudogenes were noticeably charged with high value of logFC (logFC=2.3024, P=1.55E-14 for ANXA2P2; logFC=1.308, P=5.38E-7 for ANXA2P1; logFC=1.6656, P=4.21E-11 for ANXA2P3), as well as the parent gene ANXA2 (logFC=2.435, P=1.01E-15). Therefore, we chose ANXA2 and its pseudogenes for further study.

### Expression of ANXA2 and its pseudogenes in glioma datasets

**Figure 2:**
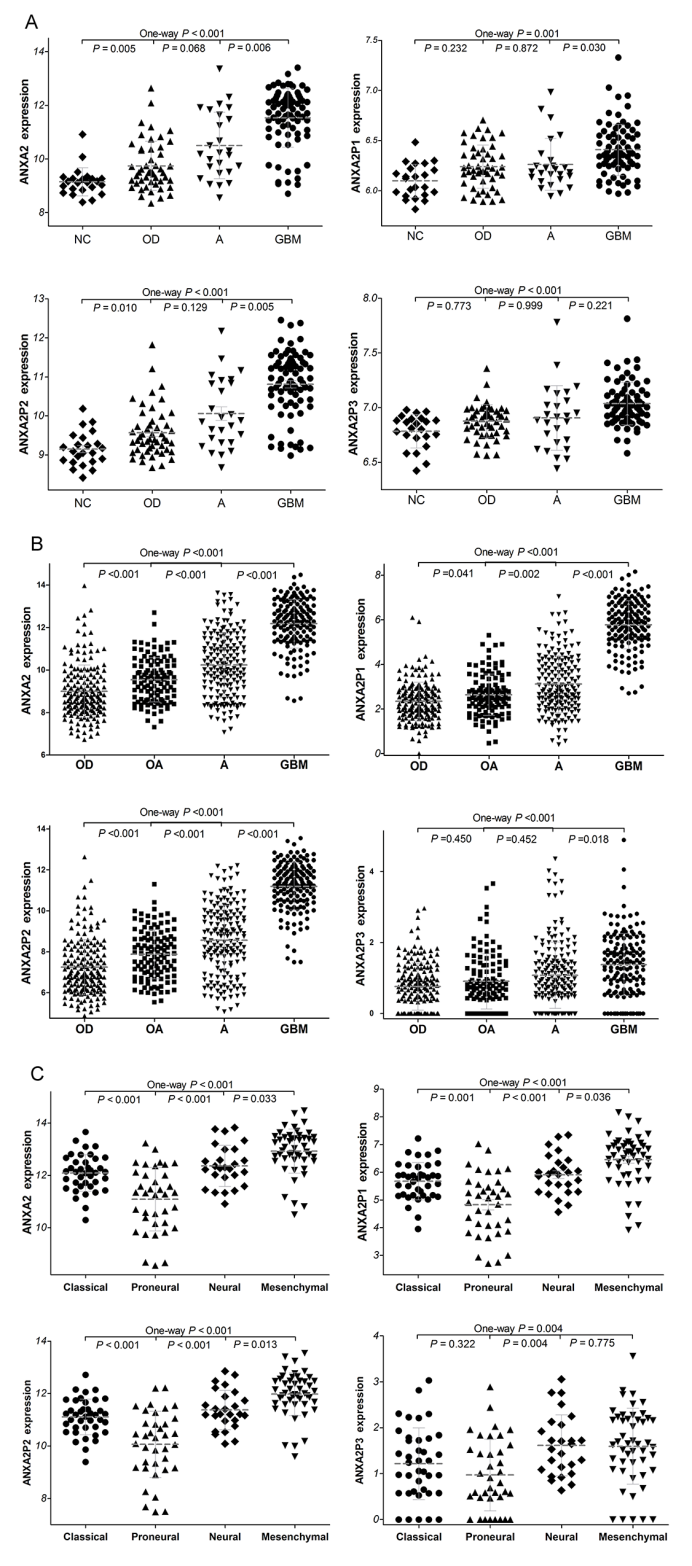
The expression of ANXA2 and ANXA2 pseudogenes in diffuse gliomas **(A)** The ANXA2 and its pseudogenes expression were analyzed in glioma samples of the GSE4290 dataset. GBM vs OD, P<0.001 for ANXA2, P=0.004 for ANXA2P1 and P<0.001 for ANXA2P2. (50 cases of OD, 26 cases of A, 77 cases of GBM and 23 cases of NC). **(B)** ANXA2 and ANXA2 pseudogenes expression in glioma samples of TCGA dataset. (175 cases of OD, 115 cases of OA, 170 cases of A and 152 cases of GBM.) Abbreviations: NC: non-tumor, OD: oligodendroglioma OA: oligoastrocytoma, A: astrocytoma, GBM: glioblastomas. **(C)** The expression of *ANXA2* and ANXA2 pseudogenes in four glioblastome subtypes of TCGA dataset. Mesenchymal subtype vs classical subtype: *P*<0.001 for ANXA2, ANXA2P1 and ANXA2P2, *P*=0.164 for ANXA2P3. (39 cases of classical subtype, 37 cases of proneural subtype, 26 cases of neural subtype, 49 cases of mesenchymal subtype)

To investigate the significance of ANXA2 and ANXA2 pseudogenes in diffuse gliomas, we have analyzed the gene expression profiles of glioma samples in GSE4290 that is the independent glioma gene expression dataset. As shown in Figure 2A, ANXA2 and its pseudogenes expression were remarkably increased in glioma tissues compared with non-tumor controls (all *P*<0.001). GBM presented a significant higher expression of ANXA2, ANXA2P1 and ANXA2P2 than astrocytoma (A, *P*=0.006, *P*=0.030 and *P*=0.005, respectively) and oligodendroglioma (OD, *P*<0.001, *P*=0.004 and *P*<0.001, respectively). Next, we performed further analysis on TCGA dataset, which verified our results in GSE4290. The expression of ANXA2 and its pseudogenes were markedly higher in GBM samples than various low-grade gliomas (LGG, all *P*<0.001; Figure 2B) that refer to some diffuse gliomas (astrocytoma, oligoastrocytoma (OA) and oligodendroglioma). However, there was no significant difference about the expression of ANXA2P3 in different types of glioma. We also performed analysis on 43378 dataset (Supplementary Figure 1). All above findings implied that ANXA2 and its pseudogenes may play an important role in the progression of diffuse gliomas.

The TCGA network defines the classical, neural, proneural, and mesenchymal subtypes of GBM by gene expression-based molecular stratification [18]. We described the expression of ANXA2 and ANXA2 pseudogenes in GBM subtypes based on the classification criterion of above. Our analyses showed that there were significant differences in ANXA2 and ANXA2 pseudogenes expression among the four GBM subtypes in TCGA dataset (*P*<0.001, *P*<0.001, *P*<0.001 and *P*=0.004, respectively; Figure 2C). Especially, ANXA2, ANXA2P1 and ANXA2P2 were preferentially expressed in mesenchymal subtype (All P<0.001) and less expressed in proneural subtype (All P≤0.001), as compared with classical subtypes. By definition, mesenchymal subtype is associated with hemizygous deletion of 17q11.2 region and expression of mesenchymal markers such as CHI3L1 and MET, and proneural subtype correlates with amplification of EGFR and lack of CDKN2A [19]. The results indicated ANXA2, ANXA2P1 and ANXA2P2 may be related to molecular markers of mesenchymal and proneural subtypes of glioblastomas.

### The correlation between ANXA2 expression and the expression of ANXA2 pseudogenes

**Figure 3:**
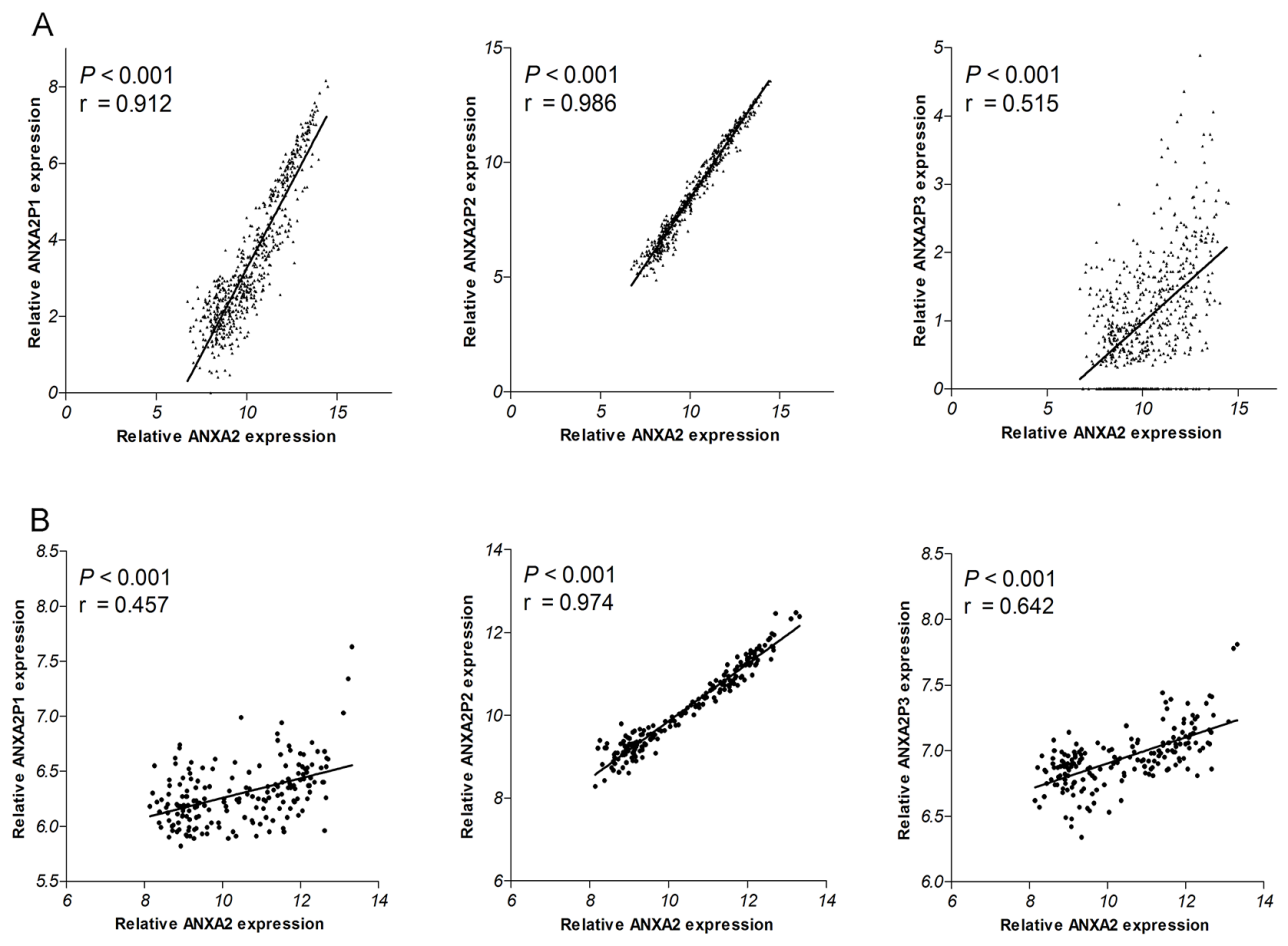
Correlations of ANXA2 expression with ANXA2 pseudogenes expression in TCGA dataset **(A)** and 4290 dataset **(B).**

In addition, Pearson’s correlation analysis was used to study the relationship between ANXA2 expression and ANXA2 pseudogenes expression in TCGA (all P<0.001; r=0.912, r=0.986 and r=0.515 respectively; Figure 3A) and GSE4290 dataset (all P<0.001; r=0.457, r=0.974 and r=0.642 respectively; Figure 3B). It was found that the expression level of ANXA2 was positively correlated with ANXA2 pseudogenes expression. These results implied that the expression of ANXA2 might be at least partly regulated by ANXA2 pseudogenes.

### Clinical validation of the ANXA2 and ANXA2 pseudogenes expression in diffuse glioma tissues

**Figure 4:**
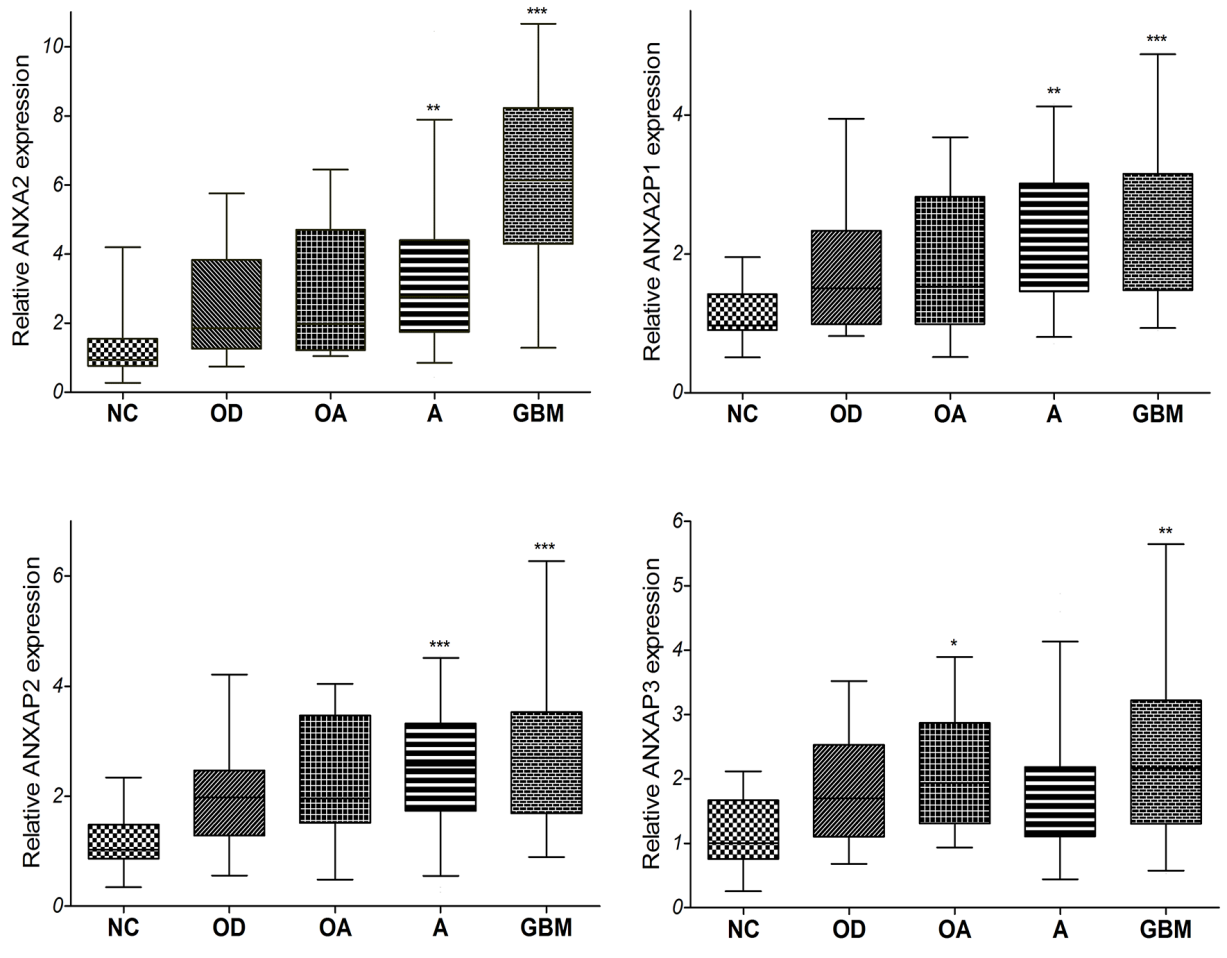
The qRT-PCR analysis of relative ANXA2 and ANXA2 pseudogenes expression level in 99 cases of tissues from diffuse glioma patients and 12 cases of non-tumor brain tissues (13 cases of OD, 10 cases of OA, 50 cases of A, 26 cases of GBM and 12 cases of NC. ^*^: P<0.05, ^**^: P<0.01, ^***^: P<0.001) Abbreviations: NC: non-tumor, OD: oligodendroglioma OA: oligoastrocytoma, A: astrocytoma, GBM: glioblastomas.

To confirm the ANXA2 and its pseudogenes expression with clinical specimens, we detected the expression levels of ANXA2 and its pseudogenes in 99 diffuse glioma specimens and 12 non-tumor brain tissues by qRT-PCR. The primary clinical characteristics of these glioma patients were listed in Supplementary Table 2. As compared with non-tumor tissues, diffuse glioma, espeically glioblastoma (Figure 4), displayed statistically higher expression level of ANXA2 and its pseudogenes. However, there are no significant differences among low-grade gliomas (oligodendroglioma, oligoastrocytoma and astrocytoma). Similar to the above analysis results of some datasets, these results indicated that ANXA2 and its pseudogenes are highly expressed in diffuse gliomas.

### Associations of the ANXA2 and ANXA2 pseudogenes expression with survival of glioma patients

**Figure 5:**
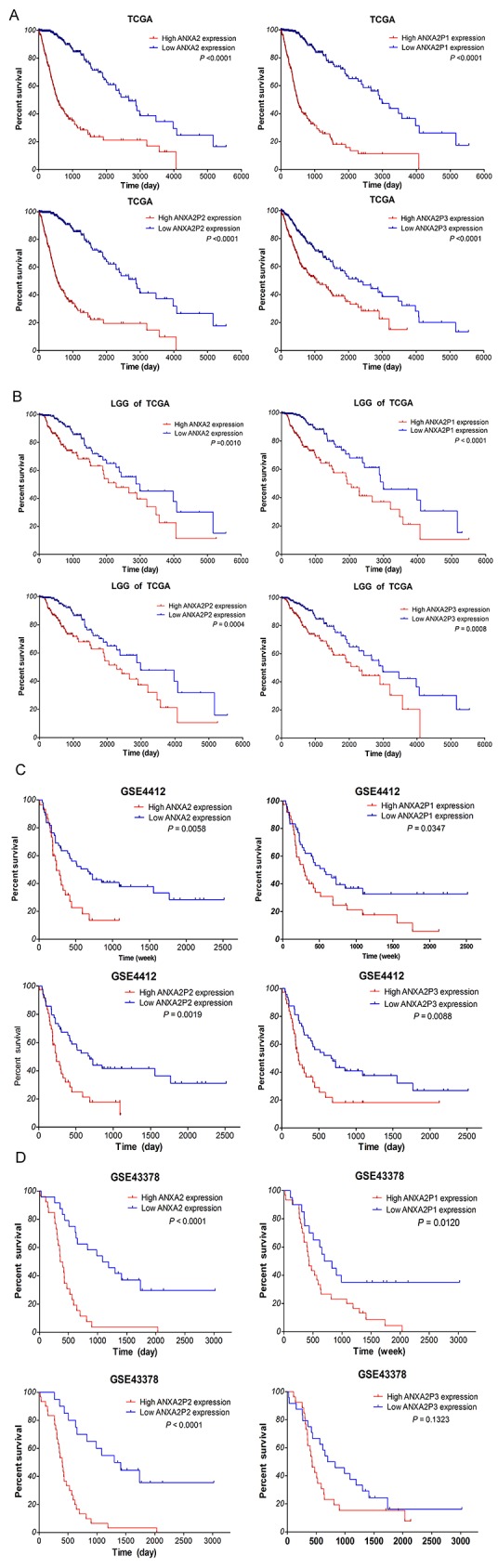
Highly expressed ANXA2 and ANXA2 pseudogenes associated with poor OS for glioma patients Kaplan–Meier survival curve analyses with a log-rank comparison were performed according to ANXA2 and its pseudogenes expression level in dataset. **(A)** The expression of ANXA2 and ANXA2 pseudogenes associated with OS for glioma patients in TCGA dataset (612 cases). **(B)** The ANXA2 and ANXA2 pseudogenes expression associated with the OS for LGG glioma patients of TCGA (460 cases). **(C-D)** The expression of ANXA2 and ANXA2 pseudogenes associated with the OS for glioma patients in GEO dataset (GSE4412:85 cases; GSE43378: 50 cases).

Next, to investigate the connections between the gene expressions with the OS, we analyzed the independent glioma gene expression data of TCGA and GEO datasets by Kaplan–Meier analysis and a log-rank comparison. The glioma patients were divided into low expression group and high expression group using the mean value of gene expression as the cutoff point. As shown in Figure 5A, analysis of TCGA dataset, the OS of glioma patients with high expression of ANXA2 and its pseudogenes were significantly worse than that with low expression (all P<0.0001). Moreover, to analyze the LGG subtypes of TCGA samples, the LGG patients with low expression of ANXA2 and its pseudogenes had better OS than that with low expression (*P*=0.0010, *P*<0.0001, *P*<0.0004, *P*=0.0008, respectively; Figure 5B). Additionally, in GSE4412 dataset, the OS of glioma patients with high expression of ANXA2 and its pseudogenes were noticeably worse than that with low expression (*P*=0.0058, *P*=0.0347, *P*=0.0019, *P*=0.0088, respectively; Figure 5C), and even more significant results were obtained from GSE43378 datasets (*P*<0.0001, *P*=0.0120, *P*<0.0001, *P*=0.1323, Figure 5D).

Furthermore, the Univariate Cox regression analysis of the OS of glioma samples within TCGA showed that high expression of ANXA2 and its pseudogenes (*P*<0.001), increasing age (*P*<0.001), high karnofsky performance score (KPS; *P*<0.001), WHO grade (II/IV, III/IV; both *P*<0.001), and histology (A/GBM, OD/GBM, OA/GBM; all *P*<0.001) were the factors associated with prognosis. Subsequent multivariate analysis results revealed that in addition to increasing age, KPS, grade and histology, high expression of ANXA2, ANXA2P1 and ANXA2P2 (HR: 1.883, *P*=0.002; HR: 2.444, *P*<0.001; HR: 2.053, *P*=0.001) were also the independent prognosis factors for survival of glioma samples (Table 1A and 1B). Cox regression analyses were also performed within the GSE43378 dataset (data not shown), the results were consistent with TCGA dataset.

All together, these results suggested that highly expressed ANXA2 and ANXA2 pseudogenes are linked with the poor survival outcome of diffuse glioma patients and ANXA2, ANXA2P1 and ANXA2P2 could be the independent prognosis factors for diffuse glioma patients.

**Table 1A:** Cox regression analyses of ANXA2 and ANXA2P1 in TCGA dataset

TCGA	ANXA2				ANXA2P1			
Variables	Univariable model		Multivariable model		Univariable model		Multivariable model	
	HR	P value	HR	P value	HR	P value	HR	P value
**High expression**	5.602	<0.001	1.883	0.002	7.234	<0.001	2.444	<0.001
**Gender, female/male**		0.646				0.646		
**Increasing age**	1.077	<0.001	1.031	<0.001	1.077	<0.001	1.032	<0.001
**KPS >80**	0.942	<0.001	0.969	<0.001	0.942	<0.001	0.969	<0.001
**Grade II/IV**	0.050	<0.001	0.162	<0.001	0.050	<0.001	0.229	<0.001
**Grade III/IV**	0.147	<0.001	0.419	<0.001	0.147	<0.001	0.506	<0.001
**Histology**								
**A/GBM**	0.141	<0.001		0.115	0.141	<0.001		0.101
**OD/GBM**	0.071	<0.001		0.054	0.071	<0.001		0.096
**OA/GBM**	0.087	<0.001		0.635	0.087	<0.001		0.958

Abbreviations: HR, hazard ratio; KPS, karnofsky performance score.

**Table 1B:** Cox regression analyses of ANXA2P2 and ANXA2P3 in TCGA dataset

TCGA	ANXA2P2				ANXA2P3			
Variables	Univariable model		Multivariable model		Univariable model		Multivariable model	
	HR	P value	HR	P value	HR	P value	HR	P value
**High expression**	6.202	<0.001	2.053	0.001	2.141	<0.001		0.429
**Gender, female/male**		0.646				0.646		
**Increasing age**	1.077	<0.001	1.032	<0.001	1.077	<0.001	1.036	<0.001
**KPS >80**	0.942	<0.001	0.970	<0.001	0.942	<0.001	0.967	<0.001
**Grade II/IV**	0.050	<0.001	0.172	<0.001	0.050	<0.001	0.420	0.001
**Grade III/IV**	0.147	<0.001	0.458	<0.001	0.147	<0.001	0.578	0.002
**Histology**								
**A/GBM**	0.141	<0.001		0.056	0.141	<0.001	0.464	0.001
**OD/GBM**	0.071	<0.001		0.034	0.071	<0.001	0.209	<0.001
**OA/GBM**	0.087	<0.001		0.730	0.087	<0.001	0.325	<0.001

### Survival outcome evaluation of patients with chemotherapy and radiotherapy

**Figure 6:**
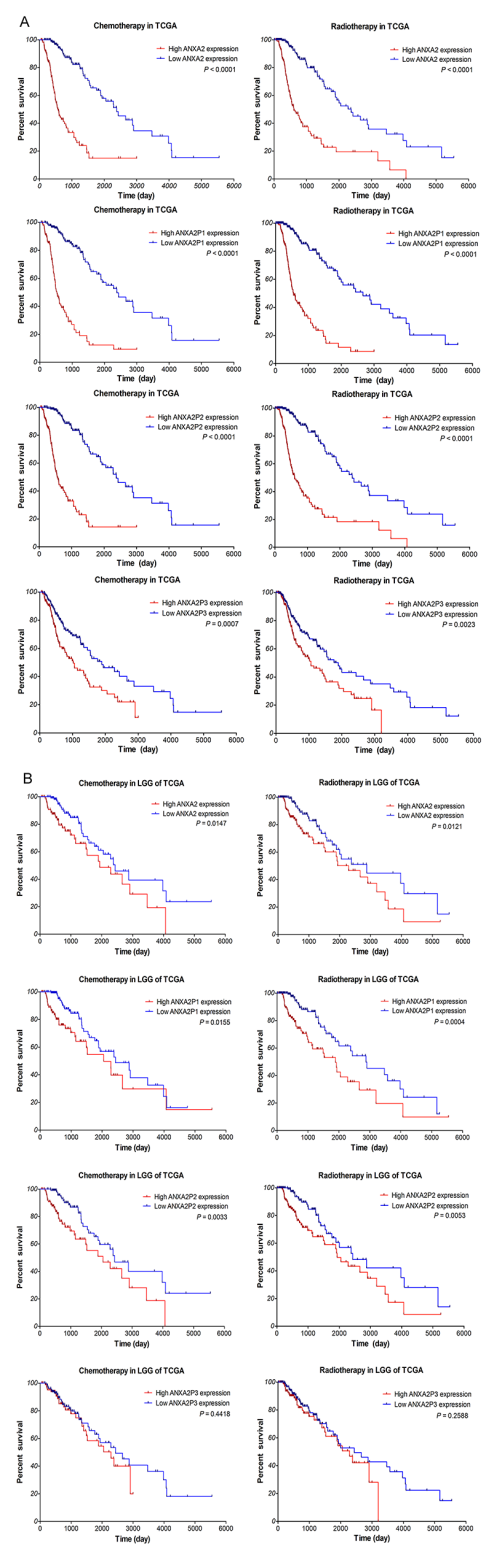
Kaplan–Meier survival curve analysis of glioma patients with chemotherapy or radiotherapy based on the expression of ANXA2 and ANXA2 pseudogenes The p-values were computed by the log-rank test. **(A)** In TCGA dataset. (390 patients with chemotherapy, 332 patients with radiotherapy). **(B)** In LGG samples of TCGA dataset. (278 patients with chemotherapy, 218 patients with radiotherapy)

To determine the associations between the ANXA2 and ANXA2 pseudogenes expression signature in response to chemotherapy and radiotherapy, subset analyses were performed with TCGA dataset, from which therapeutic information is available. As we all know that temozolomide (TMZ) chemotherapy and radiotherapy are the main therapy of the glioma patients. As shown in Figure 6A, our analyses displayed that the therapeutic outcome of TCGA patients who have received chemotherapy or radiotherapy with low expression of ANXA2 and ANXA2 pseudogenes was remarkably better than that with high expression (*P*≤0.0007, *P*≤0.0023). Also, in LGG samples (Figure 6B), patients with low-ANXA2, ANXA2P1 and ANXA2P2 expression had noticeably better therapeutic outcome than that with high expression, who have received chemotherapy (*P*=0.0147, *P*=0.0155, *P*=0.0033, respectively) or radiotherapy (P=0.0121, P=0.0004, P=0.0053, respectively). These results suggested that glioma patients with low expression of ANXA2 and ANXA2 pseudogenes could benefit more from chemotherapy and radiotherapy, and highly expressed ANXA2, ANXA2P1 and ANXA2P2 could be an indication of poor response to adjuvant therapy.

## DISCUSSION

In this study, we first identified 51 upregulated pseudogenes and 22 downregulated pseudogenes in glioblastomas based on gene expression profiles, and focused on the pseudogenes of ANXA2 (ANXA2-P1, ANXA2-P2 and ANXA2-P3) for further study. Results showed that the expression of ANXA2-P1, ANXA2-P2, ANXA2-P3 and ANXA2 was significantly up-regulated in diffuse glioma. Meanwhile, among the four glioblastoma subtypes, ANXA2P1, ANXA2P2 and ANXA2 were found to be preferentially expressed in mesenchymal subtype and less expressed in proneural subtype. In addition, Pearson’s correlation analysis found that the expression level of ANXA2 was positively correlated with ANXA2 pseudogenes expression. Furthermore, Kaplan–Meier curve and Cox regression analyses showed that the high expression of ANXA2 and its pseudogenes were correlated with the poor OS of glioma patients, and the aberrantly expressed ANXA2, ANXA2P1 and ANXA2P2 were the independent prognosis factors for gliomas. Taken together, these results suggest that three pseudogenes of ANXA2 may be suitable biomarkers for diagnostic or therapeutic strategies for diffuse gliomas.

ANXA2 gene encodes a calcium-dependent phospholipid-binding protein, which involves in processes of pathology and physiology of many neoplasms. In glioma, the expression of ANXA2 was higher in tumor tissue than in non-neoplastic tissues, and was correlated with mesenchymal and metastatic phenotype of glioblastomas [20]. ANXA2 expression was also positively correlated with glioma histologic grades and patients’ survival [21–24]. In addition, the over-expression of ANXA2 was attributed to glioma angiogenesis, migration and invasion *in vitro* [20, 24, 25] rather than proliferation and adhesion [26]. Tracing glioma progression in rodent brain, researchers found that the ANXA2 knockdown group were tumor size decreased and tumor progression slowed [23]. According to above findings, ANXA2 might serve as an independent prognostic biomarker for glioma patients. This is the same as what we found.

ANXA2-P1, ANXA2-P2 and ANXA2-P3 were three pseudogenes of ANXA2, which located on chromosomes 4, 9 and 10, respectively. Through integrated analysis of several ovarian cancer datasets, a 19-gene model which including ANAX2P1 and ANXA2P3 can serve as a reproducible predictor of survival [27]. Based on a pseudogene-mining approach, high risk scores of six-pseudogene signature (including ANXA2P3) predicted poor OS of glioma patients [28]. However, the reports about pseudogenes of ANXA2 were very limited and no one has researched ANXA2-P1, ANXA2-P2 and ANXA2-P3 independently. In this study, this is the first time that we identified the expression pattern and underlying clinical significance of ANXA2-P1, ANXA2-P2 and ANXA2-P3 respectively. Pseudogenes of ANXA2 might play important roles in diffuse gliomas progression.

Pseudogenes have been considered as non-functional relics early, however, emerging studies revealed that some pseudogenes play crucial roles in human cancer. High expression of PTENP1 might result in better OS and disease-free survival rates of HNSCC patients [29]. Low POU5F1P4 level was found to be significantly correlated with better prognosis of hepatocellular carcinoma patients [30]. DUXAP8 is a transcribed pseudogene that upregulated in non-small-cell lung cancer tissues and exhibited poor OS [31]. In this study, for the first time, we found that high expression of ANXA2 pseudogenes were correlated with the poor OS of glioma patients. What is the underlying mechanism of pseudogenes play their roles in cancer progression? Pseudogene PTENP1 exerted a growth-suppressive role in tumor through a regulatory function of PTEN, which as a decoy for PTEN-related miRNAs such as miR-17, miR-19b, and miR-20a and competed with these miRNAs [13–15]. POU5F1P4 functions as a ceRNA to protect POU5F1 transcript from being inhibited by miR-145, promoting HCC cell growth and the tumorigenicity [30]. Oncogenes, HMGA1P6 and HMGA1P7, elicit oncogenic activity as decoys of HMGA1-targeting miRNAs that elevate HMGA1 expression [32]. In present study, we first time clarified that the expression level of ANXA2 was positively correlated with ANXA2 pseudogenes expression. Like above pseudogenes and its parent genes, according to the high-homology region within gene sequence, same expression patterns, and the clinical significance among ANXA2 pseudogenes and ANXA2, one can speculate that ANXA2 pseudogenes function mechanisms in tumor progression are similar to the pseudogenes that mentioned above. The ANXA2 pseudogenes take on their roles might through regulating the expression of ANXA2. Further researches to validate the prediction and mechanisms of ANXA2 pseudogenes are on the way.

Finally, we evaluated the associations between the pseudogenes expression and the responses of chemotherapy and radiotherapy. It was found that glioma patients with low ANXA2 and ANXA2 pseudogenes expression could benefit more from chemotherapy and radiotherapy. Summarizing other studies, ANXA2 was upregulated in gastric cancer tissues and as a direct target of miR-101, miR-101 alleviated chemoresistance of cisplatin or vincristine in gastric cancer cells by targeting ANXA2 [33]. The protein ANXA2 could be bind to a well-known multidrug-efflux transporte named P-gp, which promoted the invasiveness of multidrug-resistant breast cancer cells through regulation of ANXA2 phosphorylation [34]. The expression of ANXA2 was extremely higher in recurrent Adamantinomatous Craniopharyngioma (AdaCPs) and ANXA2+ AdaCP cells were more sensitive to tyrosine kinase inhibitor gefitinib [35]. In gliomas, ANXA2 was down-regulated in glioma cells induced by docosahexaenoic acid (DHA) [36]. Anti-tumor compound, Chlorotoxin (CTX) and TM601, have been proved to be bound with cell surface ANXA2 in malignant gliomas [37, 38]. These results implied that ANXA2 and its pseudogenes could be used as potential indicators for therapeutic efficiency of glioma patients.

## MATERIALS AND METHODS

### Bioinformatics approach and databases

#### Differential expression analysis

The raw data of GSE4290 was preprocessed by affy package of Bioconductor R (www.bioconductor.org/help/workflows/arrays/) [39], and probe annotation was performed using an annotation file supplied by Affymetrix. Following normalization, the differential expression analysis between glioblastoma samples and non-tumor controls of GSE4290 dataset was performed with the Limma (Linear Models for Microarray Analysis) package of Bioconductor R (http://www.bioconductor.org/packages/release/bioc/html/limma.html) [40]. An absolute log2 fold change (|logFC|) more than 1 and the P-value adjusted by the Benjamini–Hochberg method [41] of less than 0.05 were set as cut-off criteria. Hierarchical clustering of 73 DEPs was performed by R based on the gene expression values of each sample to testify the pseudogene expression difference between glioblastoma samples and non-tumor controls. The identified DEPs including volcano plots and heat-map was visualized by the ggplot2 (http://ggplot2.org/) and gplots packages (https://cran.r-project.org/web/packages/gplots/) of R, respectively.

#### Glioma gene expression datasets

The Cancer Genome Atlas (TCGA) gene expression data (Illumina HiSeq) plus clinical information for GBM and LGG (low grade glioma) samples were obtained from TCGA data portal (www.cancergenome.nih.gov). The data were processed, normalized, matched and analyzed with customized R scripts (www.r-project.org). The dataset contained 152 GBM and 460 LGG patients, and most of them received TMZ chemotherapy and/or radiation therapy. The gene expression profiles of GSE4290 [42], GSE4412 [43], and GSE43378 [44] was downloaded from the Gene Expression Omnibus database (GEO, http://www.ncbi.nlm.nih.gov/geo/) [45]. Sample information were presented in Table 2. The original CEL files and annotation files of the platform were also downloaded. The gene expression microarrays are based on Affymetrix Human Genome U133 Plus 2.0 Array platform (Affymetrix Inc., Santa Clara, CA, USA).

**Table 2:** Clinical information of glioma samples in TCGA and GEO datasets

Datasets	GSE4290	GSE4412	GSE43378	TCGA
**Sample number (n)**	176	85	50	612
**Non-tumor sample**	23			
**Oligodendroglioma**	50	11	7	175
**Oligoastrocytoma**		7		115
**Astrocytoma**	26	8	11	170
**Glioblastoma(grade IV)**	77	59	32	152
**WHO grade II**	45		5	218
**WHO grade III**	31	26	13	242
**Gender, F / M**		53 / 32	16 / 34	257 / 355
**Age, year**		44.38±1.678	52.72±2.426	47.29±0.620
**KPS score**			Yes	Yes
**survival data**	No	Yes	Yes	Yes

Abbreviations: F, female; M, male; KPS, karnofsky performance score.

### Experimental method and materials

#### Clinical specimens

For this study, Glioma tissue specimens (n=99) were obtained from patients diagnosed with gliomas undergoing surgical resection at the Department of Neurosurgery of Xiangya Hospital of Central South University from July 2015 to August 2016. The patients were not exposed to radiation or chemotherapy before the surgery. After excision, tissue specimens were immediately frozen in liquid nitrogen for subsequent use. All clinical pathological data were assembled according to the classification of 2016 CNS WHO. Twelve non-neoplastic brain tissues were obtained from adult patients with craniocerebral injuries, which required partial resections of brain tissue as decompression treatment to reduce intracranial pressure. This study has been approved by the Ethics Committees of Central South University and the patients have provided written informed consent.

#### RNA extraction and qRT-PCR analysis

Total RNA was extracted from tissues using the TRIzol reagent (Invitrogen) following the manufacturer’s instructions. RNA concentration and quality were determined with UV spectrophotometer analysis at 260 nm and RNA quality was checked by electrophoresis. One microgram total RNA of each sample was reverse transcribed into cDNA in a final volume of 20 μl under standard conditions by using PrimeScript^RT^ reagent Kit with gDNA Eraser (Takara, Code No.RR047A). The synthesized cDNA was stored in −80°C for subsequent analysis.

Quantitative real-time polymerase chain reaction (qRT-PCR) was performed with the SYBR^®^ Premix DimerEraser™ (Takara, Code No.RR091A) on the LightCycler^®^ 480 system (Roche Diagnostics) according to manufacturer’s protocol. The qRT-PCR reaction included an initial denaturation step at 95°C for 30 s, followed by 40 cycles of 92°C for 5 s, 55°C for 30 s and 72°C for 30 s. All reactions were run in triplicate using genes specific primers. ACTB (β-actin) was used as the internal control for data normalization. Relative quantification of gene expression was calculated by the comparative cycle-threshold (CT) (2^-ΔΔCT^) method.

Primers for ANXA2, ANXA2P1, ANXA2P2 and ANXA2P3 were designed and synthesized by Sangon Biotech (Shanghai, China). Human ACTB internal control primer (B661102-0001) was purchased from Sangon Biotech. The primer sequences were supplied in Table 3.

**Table 3:** Primer sequences used for real-time PCR

Gene	Sequence
**ANXA2**	F: TGCCTTCGCCTACCAGAGAA R: GCCCAAAATCACCGTCTCC
**ANXA2P1**	F: GTGGTGGAGATGGCTGAAGTC R: CTCACAGGAACAGCCACAGGGA
**ANXA2P2**	F: CATTGTGACCAACCGCGACA R: GCCCAAAATCACCGTCTCCAG
**ANXA2P3**	F: CATCCAGCAAGACACTAAGAA R: CTATGTTGGGCTTCAGTCATC

#### Statistical analysis

The statistical analyses were performed with the SPSS 22.0 software (IBM SPSS, Chicago, USA). GraphPad Prism version 5.0 (GraphPad Software, Inc., La Jolla, USA) was used for graphing and analysis. Data are exhibited as means ± standard deviation (SD). Statistical significance of differences between two groups was assessed using two-sided Student’s *t* test; and comparisons of multiple groups were made with one-way analysis of variance (ANOVA). *P* values less than 0.05 were considered a statistically significant difference. Survival analysis was performed by the Kaplan–Meier method with the log-rank (Mantel-Haenszel) test. The risk association of gene expression among several known risk factors was determined using univariate and multivariate Cox regression analyses.

## CONCLUSION

In summary, this study demonstrated that ANXA2 pseudogenes and ANXA2 were up-regulated in diffuse gliomas tissue compared to normal brain tissue, and preferentially expressed in mesenchymal subtypes of glioblastoma. Highly expressed ANXA2 and its pseudogenes were associated with poor survival, and could be the independent prognosis factors for glioma patients. Additionally, down-regulation of ANXA2, ANXA2P1 and ANXA2P2 might contribute to the improvement of the OS for glioma patients who received chemotherapy and radiotherapy. These results implied that ANXA2 pseudogenes and ANXA2 could be potentially used as prognosis biomarkers and therapeutic targets for gliomas.

## SUPPLEMENTARY MATERIALS FIGURE AND TABLES




