# Supplementary material for: Pseudogenes of annexin A2, novel prognosis biomarkers for diffuse gliomas
Source: Oncotarget. 2017 Oct 31;8(63):106962–75. doi: 10.18632/oncotarget.22197 (PMC5739788; doi:10.18632/oncotarget.22197)
Supplement: Supplementary file 1 [file oncotarget-08-106962-s001.pdf]

Pseudogenes of annexin A2, novel prognosis biomarkers for diffuse gliomas

SUPPLEMENTARY MATERIALS

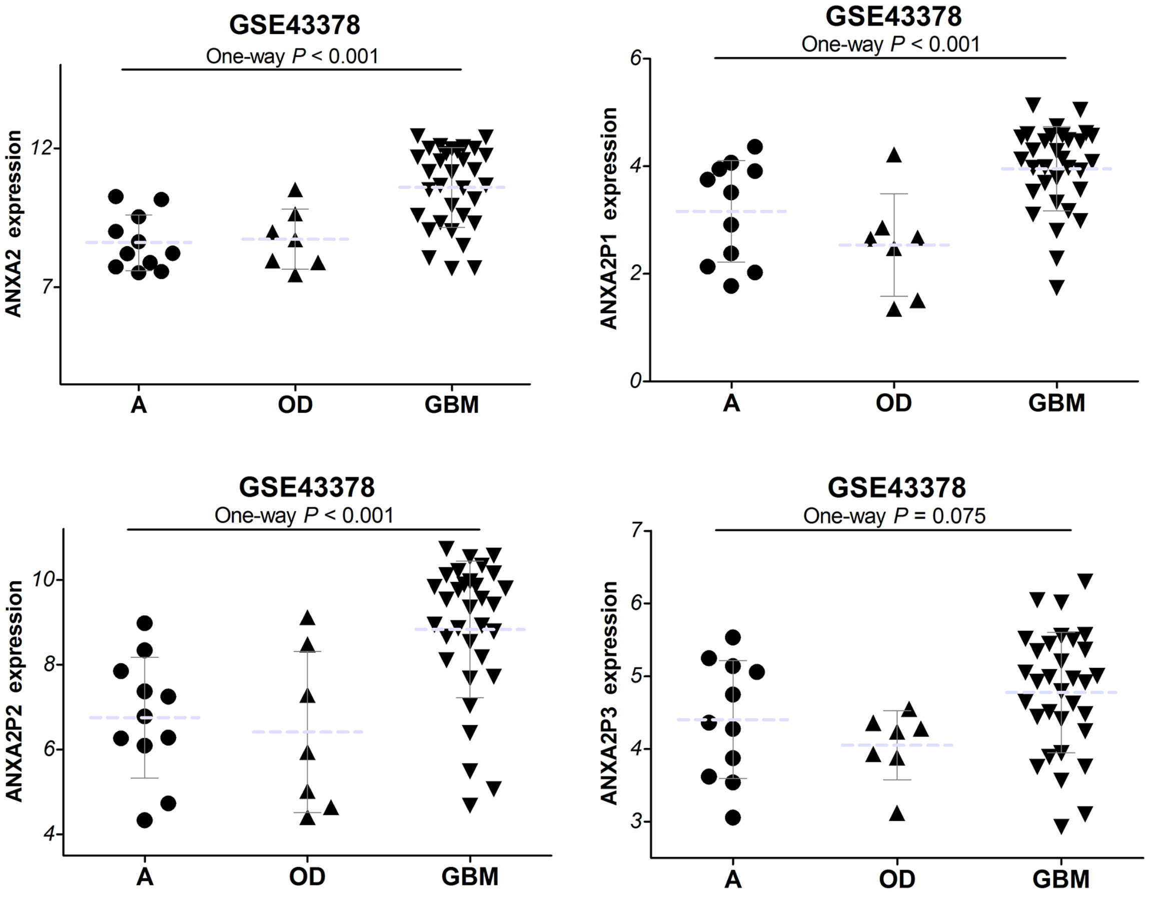

Supplementary Figure 1: ANXA2 and annexin A2 pseudogenes expression in glioma samples of the GSE43378 dataset. Total 50 cases of glioma samples (11 cases of A, 7 cases of OD, 32 cases of GBM).

Supplementary Table 2: Clinical and molecular pathology characteristics of diffuse glioma patients

Clinical characteristics	Specimens
**Number of patients (n)**	99
**Oligodendroglioma**	13
**Oligoastrocytoma**	10
**Astrocytoma**	50
**Glioblastoma with grade IV**	26
**Gender, female / male**	37 / 62
**Age at diagnosis, year**	45.34±1.543
**KPS score, >80 / ≤80**	83 / 16

Abbreviations: KPS, karnofsky performance score; +: positive, -: negative.

Supplementary Table 1: Probe sets represent on Affymetrix HG-U133 Plus 2.0 arrays.

See Supplementary File 1
